# A multi-level test of the seed number/size trade-off in two Scandinavian communities

**DOI:** 10.1371/journal.pone.0201175

**Published:** 2018-07-27

**Authors:** Amparo Lázaro, Asier R. Larrinaga

**Affiliations:** 1 Global Change Research Group, Mediterranean Institute for Advanced Studies (UIB-CSIC), C/ Miquel Marqués 21, Esporles, Balearic Islands, Spain; 2 eNeBaDa, Rúa das Penas 57, Santiago de Compostela, A Coruña, and Misión Biológica de Galicia (CSIC), Carballeira 8, Salcedo, Pontevedra, Spain; University of Wyoming College of Arts and Sciences, UNITED STATES

## Abstract

Seed size is a fundamental life-history trait for plants. A seed number/size trade-off is assumed because the resources invested in reproduction are limited; however, such a trade-off is not always observed. This could be a consequence of the method used for testing it, where the null hypothesis is dictated by common statistical practice, rather than being based on any underlying theory. Alternatively, there might be some population- and species-dependent variables that affect resource availability and, in turn, influence the presence and intensity of this trade-off. Using data on 42 herbs from two communities (lowland and alpine) from Southern Norway, we tested the validity of the classical linear model vs. two previously proposed models, based on resource competition, when assessing the existence of this trade-off at different levels. We also evaluated whether some species- (fruit aggregation, ovules/flower) and population-dependent (pollen limitation) variables could affect this trade-off. Classical linear modelling outperformed the other proposed functional models. Significant seed number/size relationships were negative in single-fruited species, whereas they were positive in species with infructescences of one-seeded fruits. Concordantly, fruit organization was the most influencing variable for the intra-specific trade-off in the lowland community. In the alpine community, species suffering higher pollen limitation showed more strongly negative slopes between seed size and seed number at the fruit/infructescence level. Across species, seed size and number were negatively related, although the relationship was significant in only one of the communities. No evidence of trade-off was found at the plant level. Linear models provide a flexible framework that allows coping with the variability in the seed number/size relationship. The emergence of the intra-specific relationship between seed number and size depends on species- and population-dependent variables, related to resource allocation and the pollination environment.

## Introduction

Seed size is an important life-history trait for plants because it directly affects seedling establishment, growth, survival, and the size and fecundity of adult plants [1, 2, 3, 4, 5]. Based on resource allocation principles, it is generally assumed that a trade-off between seed size and number exists, as the distribution of limited resources among several seeds involves the reduction in the amount of resources invested in each one of them [6, 7]. The seed number/size trade-off has received much attention from ecologists, mainly because the total reproductive output of plants is determined by the combination of both the quantity and quality of their seeds. Accordingly, several intra-specific studies have shown negative relationships between seed number and size at the plant [3, 8] and fruit level [3, 4, 9, 10, 11], or across populations [12, 13]. However, intra-specific studies have also shown a large number of exceptions (see review in [14]), with the number of seeds and their size often being uncorrelated [15, 16, 17, 18, 19, 20] or even positively associated [20, 21].

One of the reasons underlying the variable outcomes of intra-specific studies might be related to the particular statistical model fitted thereof, and its associated null hypotheses and assumptions. The seed number/size trade-off is usually tested by means of linear models in which seed size is linearly related to the number of seeds, despite the lack of theoretical grounds for this type of relationship. Back in 1974, Smith and Fretwell [6] assumed a constant amount of resources were to be allocated among the produced number of offspring, and formalized this assumption through a simple inverse model. In 2003, Lescourret and Génard developed a generalisation of Smith and Fretwell’s [6] model, which allows for linear and non-linear inverse relationships [22]. This generalisation is based on the negative slope between the number of units and the unit size (imposed by resource competition) and on the existence of an optimal total unit mass produced for intermediate number of units. Their model could be used at different levels of resource organization (fruit, inflorescence or plant) and was claimed to fit reasonably well for a number of wild and cultivated plant species. Despite its appeal, Lescourret and Génard’s model has not, to our knowledge, been further tested, nor has it been compared to any alternative model; and we do not yet know whether a trade-off governs the relationship in nature between seed size and number.

The contrasting results in intra-specific studies might also partly be due to the effect of several population- and species-variables on resource availability and allocation. Accordingly, the slope of the relationship between seed number and size has been related to inter-annual [23] or monthly [1, 24] changes in the availability of resources, growth conditions [25], or to the existence of symbiotic relationships [26]. However, the traits that influence the strength and direction of the relationship between seed number and size have been less explored. Multispecies tests conducted in natural communities subject to a common pollinator fauna might be useful to elucidate such traits, possibly reducing the inherent variability that exists between sites and years. In general, one could expect that population variables influencing resource availability for seeds, such as pollination intensity, may affect the relationship between seed number and size. Thus, if plant reproduction is limited by pollen receipt, instead of by resources, this trade-off (i.e. a negative relationship between seed number and size) might not appear. However, how pollen limitation affects the size-number trade-off in natural communities is still unknown. Besides, the relationship between seed number and size may also be affected by structural traits of species, such as fruit aggregation into infructescences, that influence the way resources are distributed among reproductive units, or the number of ovules per flower, which imposes a limit to the trade-off (by limiting the maximum number of seeds). A community perspective may help in the interpretation of patterns in terms of the intra-specific relationship between seed number and size, because other variables such as habitat type and weather conditions remain equal for all species considered. In addition, the study of different communities could allow for an assessment of the extent to which the factors affecting this trade-off depend on the particular ecological and evolutionary conditions of communities.

At the inter-specific level, however, the results are far more consistent and numerous studies in the literature confirm the seed number/size trade-off [27, 28, 29, 30, 31]. This inter-specific trade-off, which is normally tested as the linear relationship of log-transformed variables, has been suggested as playing an important role in the coexistence of plant species, and in the diversity and structure of plant communities [28, 29, 32, 33, 34, 35]. The evaluation of the seed number/size trade-off at both the intra- and inter-specific levels of a group of species in a locality might help shed light on the levels at which the trade-off actuates, as well as providing an understanding of the ecological and evolutionary implications thereof (within the appropriate context).

In this paper, we studied the existence of trade-offs between seed number and size both at the intra-specific and inter-specific levels, with a special interest in testing appropriate mathematical models. We used data on 42 common European herbs from two different communities from southern Norway (one lowland and one alpine community). Our specific objectives were: 1) to compare the classical statistical model commonly used to study the intra-specific relationship between seed number and size with the functional models proposed by Smith and Fretwell ([6]; S&F model, hereafter) and Lescourret and Génard ([22]; L&G model, hereafter), 2) to determine the generality of intra-specific trade-offs (i.e. negative relationships in nature) between seed number and size at different structural scales (fruit, inflorescence, and plant level), 3) to assess whether fruit organization (infructescences with multiple one-seeded fruits vs. single fruits), number of ovules per flower, or the extent of pollen limitation influence the intensity of the putative intra-specific trade-off at the fruit or infructescence level; and 4) to test the existence of a trade-off at the inter-specific level. We expected the functional L&G model to perform better than the linear and S&F models when testing the seed number/size trade-off, as it is functionally based and allows for a higher flexibility than the S&F model. We also expected pollen limitation to reduce intra-specific seed number/size trade-offs, as well as stronger trade-offs in more dependent physiological units (e.g. seeds within fruits) compared to less dependent ones (e.g, fruits within inflorescences).

## Materials and methods

### Study areas and species

We conducted our study in two different plant communities in Norway that represent typical alpine and temperate plant communities. One community is a plant and pollinator species-rich semi-natural meadow at Ryghsetra (59°44’03”N, 10°02’48”E), Buskerud county, ca 2.5 km south-east of Mjøndalen, at 300 m altitude (lowland community, hereafter). Here, the blooming season starts in early-mid May and ends in mid-late August, with ca. 55 species blooming during this period.

The other community is on a southwest exposed slope on Sandalsnuten, at Finse (60° 36’36”N, 7°31’12”E), in the northern part of Hardangervidda, alpine southwest Norway, at ca. 1450 m altitude (alpine community, hereafter). Here, the blooming season starts in late-June and ends in late-August, with ca. 25 species blooming during this period.

A total of 42 entomophilous herbs are included in this study, 24 from the lowland and 18 from the alpine community (Table 1). We selected these species because they were abundant, allowing adequate sample sizes for the analyses (see sample sizes in Tables 2 and 3), and because they are typical species of temperate and alpine meadows, and may therefore be deemed representative of these communities.

**Table 1:** Plant species of each community included in this study. Their family, the levels at which the trade-off was calculated (F: fruit; I: infructescence; P: plant), the pollen limitation indices (PL), and the average number of ovules per flowers or inflorescence are given. PL was estimated as 1- seed set of open pollinated plants / seed set of hand-pollinated plants (see [36], for details). Nomenclature follows [68].

Community	Species	Family	Levels	PL	Ovu. / fl.	Community	Species	Family	Levels	PL	Ovu./flo.
Lowland	*Anthyllis vulneraria*	Fabaceae	I, P	-0.12	1.69	Alpine	*Astragalus alpinus*	Fabaceae	F, P	0.18	4.053
	*Centaurea jacea*	Asteraceae	I, P	-0.10	74.76		*Bartsia alpina*	Scrophulariaceae	F, P	0.42	47.61
	*Centaurea scabiosa*	Asteraceae	I, P	NA	77.48		*Campanula rotundifolia*	Campanulaceae	F, P	-0.03	121.37
	*Fragaria vesca*	Rosaceae	F, P	0.01	58.49		*Cerastium alpinum*	Caryophyllaceae	F, P	0.02	42.74
	*Galium boreale*	Rubiaceae	F, P	NA	42.00		*Dryas octopetala*	Rosaceae	F, P	-0.04	59.67
	*Galium mollugo*	Rubiaceae	F, P	0.02	15.69		*Erigeron uniflorus*	Asteraceae	I, P	-0.01	139.79
	*Geranium sylvaticum*	Geraniaceae	F, P	0.21	4.28		*Gentiana nivalis*	Gentianaceae	F, P	0.02	172.91
	*Geum rivale*	Rosaceae	F, P	0.01	77.33		*Geranium sylvaticum*	Geraniaceae	F, P	0.17	4.92
	*Knautia arvensis*	Caprifoliaceae	I, P	-0.15	60.88		*Leontodon autumnalis*	Asteraceae	I, P	0.13	39.29
	*Lathyrus linifolius*	Fabaceae	F, P	0.12	9.36		*Parnassia palustris*	Celastraceae	F, P	-0.02	402.19
	*Leucanthemum vulgare*	Asteraceae	I, P	-0.12	155.13		*Pinguicula vulgaris*	Lentibulariaceae	F, P	0.07	112.35
	*Linum catharticum*	Linaceae	F, P	-0.12	9.73		*Potentilla crantzii*	Rosaceae	F, P	-0.13	27.40
	*Lotus corniculatus*	Fabaceae	F, P	0.10	29.69		*Ranunculus acris*	Ranunculaceae	F, P	0.09	23.46
	*Pilosella lactucella*	Asteraceae	I, P	0.20	42.58		*Saussurea alpina*	Asteraceae	I, P	-0.15	14.55
	*Pilosella officinarum*	Asteraceae	I, P	0.19	80.43		*Saxifraga aizoides*	Saxifragaceae	F, P	-0.18	144.79
	*Pilosella pubescens*	Asteraceae	I, P	-0.13	26.42		*Silene acaulis*	Caryophyllaceae	F, P	0.79	17.36
	*Polygala vulgaris*	Polygalaceae	F, P	0.30	2.00		*Veronica alpina*	Plantaginaceae	F, P	0.09	53.33
	*Potentilla crantzii*	Rosaceae	F, P	-0.21	37.60		*Veronica fruticans*	Plantaginaceae	F, P	-0.04	26.79
	*Potentilla erecta*	Rosaceae	F, P	-0.02	5.66						
	*Potentilla thuringiaca*	Rosaceae	F, P	-0.15	39.71						
	*Prunella vulgaris*	Lamiaceae	F, P	0.26	3.26						
	*Ranunculus acris*	Ranunculaceae	F, P	-0.03	24.91						
	*Trifolium pratense*	Fabaceae	I, P	-0.24	1.03						
	*Vicia cracca*	Fabaceae	F, P	0.63	4.89						

**Table 2:** Model comparison at the fruit or infructescence level (of one-seeded fruits). A) The lowland and B) the alpine communities. Measurement level (Level): F, fruit; I, infructescence. n: number of individuals. LIN: classic linear models; S&F: Smith and Fretwell’s model [6]; L&G: Lescourret and Génard’s model [22]. Bold letters indicate the best model for each model selection criterion. ´Res. Analysis´ indicates the best model as assessed by residual analysis (only when AICc and RRMSE best models differed); ‘ = ´ indicates very similar residual plots. Empty cells correspond to models that did not converge.

Community	Level	Species	*n*	AICc			RRMSE			Res. analysis
				LIN	S&F	L&G	LIN	S&F	L&G	
A) Lowland	F	*Fragaria vesca*	39	**69.35**	127.48	98.47	**0.21**	0.45	0.46	
		*Galium boreale*	16	**46.36**	61.86	54.89	**0.34**	0.61	0.76	
		*Galium mollugo*	78	**302.34**	410.27	400.1	**0.22**	0.44	0.23	
		*Geranium sylvaticum*	15	**62.58**	72.22	69.58	0.34	0.52	**0.33**	LIN
		*Geum rivale*	15	**76.48**	122.29	84.57	0.23	1.16	**0.15**	LIN
		*Lathyrus linifolius*	10	62.88	**61.87**	65.83	0.30	0.35	**0.05**	L&G
		*Linum catharinum*	71	**302.96**	476.48	392.81	**0.23**	0.78	2.21	
		*Lotus corniculatus*	16	**24.71**	44.31	29.65	**0.30**	0.61	1.23	
		*Polygala vulgaris*	48	**83.95**	102.83	168.51	**0.26**	0.32	0.27	
		*Potentilla crantzii*	14	**32.76**	58.76	61.37	**0.18**	0.51	1.16	
		*Potentilla erecta*	50	**208.60**	212.04	310.12	**0.17**	0.18	0.22	
		*Potentilla thuringica*	35	**159.73**	196.87	251.68	**0.16**	0.28	0.23	
		*Prunella vulgaris*	24	**98.23**	113.68	136.56	**0.20**	0.30	0.43	
		*Ranunculus acris*	22	**12.14**	54.93	39.83	**0.19**	0.54	1.29	
		*Vicia cracca*	23	**140.03**	141.91	143.64	0.22	0.24	**0.02**	=
	I	*Anthyllis vulneraria*	23	**64.32**	87.54	76.33	0.28	0.49	**0.21**	LIN
		*Centaurea jacea*	54	**83.85**	207.71	154.21	**0.17**	0.54	1.51	
		*Centaurea scabiosa*	24	**97.50**	143.21	131.26	0.23	0.64	**0.13**	LIN
		*Knautia arvensis*	13	**51.90**	73.86	57.60	**0.30**	0.79		
		*Leucanthemum vulgare*	76	**219.68**	407.05	365.69	**0.17**	0.57	0.37	
		*Pilosella lactucella*	36	**28.20**	109.52	59.98	**0.23**	0.75	1.29	
		*Pilosella officinarum*	32	**25.18**	111.63	34.53	**0.24**	0.95	1.03	
		*Pilosella pubescens*	25	**108.52**	191.06	127.48	**0.26**	1.42	1.18	
		*Trifolium pratense*	15	72.61	112.61	**70.21**	0.29	1.24	**0.14**	
B) Alpine	F	*Astragalus alpinus*	35	**67.10**	92.34	84.56	0.23	0.34	**0.22**	LIN
		*Bartsia alpina*	46	**48.12**	146.04	121.31	**0.20**	0.59	1.35	
		*Campanula rotundifolia*	27	**99.54**	146.41	127.23	**0.25**	0.61	2.38	
		*Cerastium alpinum*	28	**57.71**	128.73	73.11	**0.23**	0.86	0.68	
		*Dryas octopetala*	39	**134.23**	207.29	180.95	**0.22**	0.58	0.32	
		*Gentiana nivalis*	40	**113.21**	178.91	199.41	**0.22**	0.51	5.64	
		*Geranium sylvaticum*	19	**66.10**	79.55	97.63	**0.35**	0.53	1.55	
		*Parnassia palustre*	73	**172.86**	292.32	315.77	**0.21**	0.49	4.98	
		*Pinguicula vulgaris*	16	**25.23**	32.39	54.04	**0.24**	0.33	12.1	
		*Potentilla crantzii*	55	**181.12**	223.31	263.75	**0.19**	0.29	0.38	
		*Ranunculus acris*	42	**34.33**	92.82	60.14	**0.23**	0.48	0.85	
		*Saxifraga aizodes*	15	**50.56**	96.5	68.68	**0.22**	1.12	3.83	
		*Silene acaulis*	42	**132.67**	177.05	190.65	**0.22**	0.39	0.46	
		*Veronica alpina*	52	**118.84**	142.83	304.16	**0.12**	0.16	0.91	
		*Veronica fruticans*	15	**62.82**	97.57	77.79	**0.27**	0.95	0.37	
	I	*Erigeron uniflorus*	47	**220.37**	401.32	320.61	**0.17**	1.21	1.36	
		*Leontodon autumnalis*	45	**16.80**	57.28	29.87	**0.24**	0.39	0.76	
		*Saussurea alpina*	57	**34.77**	145.36	123.79	**0.21**	0.55	0.79	

**Table 3:** Model comparison at the plant level. A) The lowland and B) the alpine communities. *n*: number of individuals. LIN: classic linear models; S&F: Smith & Fretwell’s model [6]; L&G: Lescourret & Génard’s model [22]. Bold letters indicate the best model for each model selection criterion. ´Res. Analysis´ indicates the best model as assessed by residual analysis (only when AICc and RRMSE best models differed).

Community	Species	*n*	AICc			RRMSE			Res. analysis
			LIN	S&F	L&G	LIN	S&F	L&G	
A) Lowland	*Centaurea jacea*	19	**188.41**	199.11	197.25	0.23	0.32	**0.00**	LIN
	*Fragaria vesca*	12	**80.03**	83.73	103.99	0.30	0.41	**0.04**	LIN
	*Geranium sylvaticum*	11	65.32	65.62	**62.43**	0.27	0.33	**0.05**	
	*Geum rivale*	15	**151.05**	152.72	154.08	0.29	0.35	**0.01**	LIN
	*Knautia arvensis*	15	**178.33**	181.58	191.87	0.24	0.30	**0.002**	LIN
	*Linum catharinum*	15	**16.64**	38.64	28.80	**0.28**	0.64	1.21	
	*Pilosella pubescens*	31	**42.38**	45.04	46.34	0.32	0.41	**0.18**	LIN
	*Polygala vulgaris*	14	**42.90**	61.43	61.27	0.22	0.49	**0.11**	LIN
	*Potentilla thuringica*	15	82.95	**78.68**	81.69	0.24	0.23	**0.03**	S&F
	*Ranunculus acris*	14	**95.18**	101.64	103.86	0.25	0.36	**0.04**	LIN
B) Alpine	*Astragalus alpinus*	15	**62.17**	67.83	63.41	0.24	0.33	**0.08**	LIN
	*Geranium sylvaticum*	15	**92.26**	101.96	97.17	0.25	0.39	**0.03**	LIN
	*Potentilla crantzii*	17	50.42	51.62	**47.56**	0.21	0.24	**0.10**	
	*Saussurea alpina*	18	**124.87**	131.78	130.75	0.31	0.41	**0.06**	LIN
	*Saxifaga aizodes*	15	**65.17**	81.64	72.71	0.30	0.59	**0.18**	LIN
	*Silene acaulis*	10	**24.10**	31.52	29.12	**0.31**	0.55	0.39	
	*Veronica alpina*	13	29.40	**27.67**	31.19	**0.26**	0.28	0.34	LIN
	*Veronica fruticans*	15	**22.12**	35.31	26.68	**0.30**	0.52	0.84	

### Seed number/size trade-off at the fruit /infructescence level

At the beginning of the field season, we placed 30 permanent 2 x 2-m plots in both communities. At the lowland community, where the meadow was large and relatively homogeneous, we placed the plots in a systematic way, along two lines. At the alpine community, on the other hand, where vegetation was not homogenous across the whole area, we placed the plots haphazardly within suitable sub-areas in the community. Minimum separation between plots was 3 m in both sites.

Within these plots, during the flowering peak of each species, we haphazardly selected and marked (using a piece of drinking straw) one open flower per individual in three individuals per study species, unless fewer individuals were present in the plot (see Table 2 for the sample size of each plant species). The mean number of flowers produced per individual for each species varied from 2.25 to 30.4 at the lowland community, and from 1 to 21.4 at the alpine community. At the end of the ripening season, we collected the fruits of the marked flowers, once ripe and immediately before dispersal. The fruits were stored in paper bags until they were dissected in the laboratory in order to count the number of ovules, aborted seeds and fully developed seeds. Thereafter, we weighed (± 0.001 g) all developed seeds in a fruit together and obtained a mean seed weight per seed by dividing the total weight by the number of developed seeds in the fruit. We used seed mass as a proxy for seed size. Although having only one fruit per plant is a limitation in our study due to large variability within plants that has been sometimes reported [2, 8], we chose to maximise the number of species and the number of individuals per species rather than number of fruits per individual, taking into account the objectives of our study.

In the case of species with single-seeded fruits aggregated into inflorescences (all the Asteraceae, but also *Knautia arvensis*, *Anthyllis vulneraria* and *Trifolium pratense*), we regarded inflorescence as the lower rank reproductive unit to study, due to their particular floral arrangement. Hence, minus our marking and collecting of whole inflorescences and infructescences instead of single flowers and fruits, we followed the same protocol.

For the purposes of this study, for each species we used the lowest level at which the trade-off between seed number/size could be measured; and hereafter we distinguish between the trade-off at the fruit level (for single fruits) and at the infructescence level (for one-seeded fruits aggregated into infructescences). However, our results for infructescences containing one-seeded fruits are not necessarily extrapolable to any other type of infructescence.

### Trade-off at the plant level

To study the trade-off at the plant level, we used data from 10 to 19 individual plants haphazardly selected across the meadows (see Table 3 for the sample size of each species) from 18 of the study species (the rest of the study species only had one to three flowers or inflorescences, preventing their inclusion in this analysis). These individuals, which were different from those selected when studying the trade-off at the fruit level, were marked during the field season and all their flowers or inflorescences were counted. When the fruits were ripe, we collected separately in paper bags each of their fruits or infructescences and weighed the total seed mass produced by each of them, following the previously explained protocol. Hence, we studied the correlation between the number of fruits/infructescences and the total mass of seeds produced by each of them.

### Ovules per flower and pollen limitation

The number of ovules per flower or inflorescence (depending on the species) was calculated for each species by averaging the number of ovules counted per flower (or inflorescence) for all the individuals marked of each species.

As a measure of pollen limitation in the study plant species, we used the data on pollen limitation indices estimated by ourselves at the same study sites and during the same year [36]. These indices were based on an experiment of supplementary hand-pollination on 15 to 30 pairs of individuals/branches of each study species (see Table 1 for pollen limitation indices for each study species). One individual/branch of each pair was haphazardly assigned to receive only natural pollination (N), whereas the other was hand-pollinated (S) by depositing onto the stigmas pollen from at least five individuals situated across the study areas. We calculated an index of pollen limitation per study pair or individual as: PL_I_ = 1 –(N/S), where N is the seed set (seeds/ovules) of natural pollinated individual or branch and S is the seed set of supplementary hand-pollinated individual or branch [37]. We then averaged the indices for each pair of individuals or branches to obtain an index of pollen limitation per species (PL; see [36] for further details). Pollen limitation indices calculated at the population-level have been used in previous studies to test for inter-specific differences within communities [36, 38]. As it is a population-level index, it depends on the pollination context that a species experiences in a particular population. This implies that the relationship between this variable and the slope of the seed number/size relationship might change at a different site, even when analysing the same species.

### Statistical analyses

#### Statistical vs. functional model

We used generalised linear mixed models (GLIMMIX procedure sas v. 9.2) to compare a classical statistical approach (linear model, LIN model hereafter) to the old model proposed by Smith and Fretwell ([6]; S&F model, hereafter), and the new model proposed by Lescourret and Génard ([22]; L&G model, hereafter) based on the functional role of resource allocation.

The linear model was defined as:

M=a+b*n, (1)

S&F model [6] would be:

M=c*(1/n), (2)

whereas the L&G model [22] was defined as:

$$M=M_{\max}*(1-{\left(n/n_{\max}\right)}^{\alpha }),$$ (3)

where *M* was the mean weight of the n units (which, at the lowest level, correspond to seeds). *M*_max_ and *n*_max_ correspond to the maximum value of, respectively, *M* and *n* across the data set. Hence, a, b, c and α were the parameters describing the relationship between unit (seed) number and weight for the LIN, F&S, and L&G models, respectively. Please note that both the S&F and L&G models force the relationship to be negative, while the linear one does not. As the L&G model considers *M*_max_ as a theoretical value corresponding to n = 0, we added 0.1 to the observed maximum seed weight before including it in the model to avoid cases where *M* = *M*_max_ (which forces n to be 0).

We aimed at comparing the models by means of AICc scores (Akaike Information Criterion corrected for small sample sizes), a method that requires all the models to be fitted exactly to the same response variable, using the same statistical technique [39]. That meant that we needed to linearize the L&G model, while keeping the same response variable for all three models. In order to achieve this, we rearranged model equations using a new response variable defined as *M*_max_−*M*. The resulting equation for the linear model corresponded to:

$$y=M_{\max}-M=M_{\max}-a-b*n,$$ (4)

while for the S&F model it was:

$$y=M_{\max}-M=M_{\max}-c*(1/n),$$ (5)

and for the L&G model:

$$y=M_{\max}-M=e^{\left[\log\left(M_{\max}\right)+\alpha *\log(n/n_{\max})\right]}.$$ (6)

Using the same fitting procedure, we fitted all three model types into a GLMM framework. For the linear and S&F models, we simply fitted (4) and (5) using a Gaussian distribution and identity link function. For the L&G model we used a Gaussian distribution and a log link function in a generalised linear model where log (*M*_max_) corresponded to an offset variable and log (*n*/*n*_max_) to the explanatory variable. Hence, we fitted the following model with a log link function:

$$M_{\max}-M=\log\left(M_{\max}\right)+\alpha *\log\left(n/n_{\max}\right).$$ (7)

By doing so, instead of log-transforming the response variable and applying a simple linear model, we kept errors additive in both model types and obtained AICc values calculated at the (*M*_max_−*M*) scale, comparable to those of the linear model [40].

To select the best of the three models for each species at each level, we used both AICc [39] and root mean square (RMSE). As seed weight was highly variable among species, in this case we used the relative RMSE (RRMSE), in order to properly compare its value among species. RRMSE expresses the error as a function of average y, and it is calculated as RRMSE = RMSE / mean-y (see for example [22]). When the best model selected based on AICc and RRMSE did not match, we selected the final model based on residual analysis.

This whole process was repeated for the fruit, infructescence and plant level, following Lescourret and Génard [22]. At each level, *M* corresponds to the mean seed weight per reproductive unit and *n* corresponds to the number of units. Therefore, at the fruit level, the unit of interest is the individual seed: *M* corresponds to the mean weight of one seed produced by the fruit and *n* to the number of seeds produced by the fruit. At the infructescence level, the unit of interest is the fruit, and hence, *n* is the number of fruits and *M* is total seed weight produced per fruit (across-fruit average). As in our case all the infructescences had one-seeded fruits, the mean seed weight produced per fruit is equivalent to the mean weight of one seed produced in a fruit. At the plant level the units are either fruits or infructescences (depending on the plant species), and hence *n* corresponds to the number of either fruits or infructescences, and *M* to their total seed production in weight (across-fruit/infructescence average). For the fruit and infructescence levels, we used one unit (either fruit or infructescence) per plant to estimate seed production, while for the plant level we used data from all fruits or infructescences collected for each individual plant.

Preliminary analyses showed that the plot was a negligible source of variation (models with plot as a random effect showed higher AICcs than those without this variable, with estimates of random plot effect close to zero), and therefore, for simplicity, we did not include plot in the models shown here.

The analyses in the following sections were based on the model (linear, S&F or L&G) selected in the first section as the model best suited to properly test the relationship between seed number and size. Due to multiple testing on different species, we controlled for the false discovery rate using the Benjamini-Hochberg procedure [41].

#### Factors related to intensity of the intra-specific trade-off

We used phylogenetical generalised least squares analyses (PGLS) to study, for each community, how pollen limitation and the number of ovules per flower related to the intensity of the trade-off at the fruit/infructescence level. In the analysis for the lowland community, we also included fruit organization (single fruits vs. infructescences with multiple one-seeded fruits) as another predictor variable; however, this variable could not be included in the analyses for the alpine community because, in this community, there were only 3 species with single-seeded fruits aggregated into infructescences. The intensity of the trade-off was the response variable, which was estimated as the slope of the relationship between seed number and weight for each species. Larger negative values of this slope (lower slopes) indicate stronger trade-offs, whereas larger positive values (greater slopes) indicate strong positive relationships between seed number and weight, and, therefore, the absence of a trade-off. By using PGLS we controlled for the phylogeny of the species in the analysis of the studied correlations [42, 43]. We used Phylocom ver. 4.2 [44] and the megatree R20120829 to construct the topology and adjust the branch lengths of the phylogenetic tree of the study species in each community. PGLS analyses were conducted in R (ape [45] and nlme [46] libraries in R 3.1.2; [47]). We optimised Pagel’s lambda (λ; [48]) along with the parameters of the model to minimise the effects of wrong model selection (phylogenetically versus non-phylogenetically controlled analysis) because the suitability of our data for phylogenetic regression is, a priori, unknown [49]. The parameter λ [48] is a measure of “phylogenetic signal,” i.e. the extent to which correlations in traits reflect their shared evolutionary history (as approximated by Brownian motion). A λ value of 0 indicates phylogenetic independence of species’ traits, a value of 1 indicates that species’ traits covary in direct proportion to their shared evolutionary history (Brownian motion model), and a value > 1 indicates a phylogenetical signal higher than the one expected under the Brownian motion model [50]. Prior to the analyses, we standardised the regression slopes by multiplying them by the quotient of the standard deviation of the predictor and the standard deviation of the response variable [51]. We used AICc (library sme; [52]) to select best models among the set of combinations of predictor variables.

#### Trade-off at the inter-specific level

To test for the seed number/size trade-off at the among-species level, we also used phylogenetical generalised least squares analyses (PGLS), optimising Pagel’s lambda (λ; [48]) along with the parameters of the model. However, in this case we related species’ average number of seeds per fruit (or infructescence) to species’ average seed weight, using the data of one fruit/infructescence per plant. Prior to the analyses, variables were log-transformed to achieve normality.

## Results

### Statistical vs. L&G and S&F models

Overall, the classical statistical linear model was most often selected as the best model; however, the results varied among both species and structural levels.

At the fruit or infructescence level, AICc values showed that linear models were better than the other models explaining the trade-off for all the species, except for one species for which the S&F model was slightly better (*Lathyrus linifolius*; Table 2), and another for which the L&G model was better (*Trifolium pratense*; Table 2). The results based on RRMSE in general agreed with those based on AICc, except for 7 species (Table 2). One of these species was *Lathyrus linifolius*, for which the residual analysis showed L&G to be the best model (Table 2). A residual analysis of the models of the other 6 species showed that linear models offered a better fit (all cases but one, for which residuals of both models -linear and L&G- were similar; Table 2).

Also, at the plant level linear models were more consistently selected as the best models, although the results were not so clear-cut at this level. Only for 5 species AICc and RRMSE matched in the selected best model, which corresponded to the L&G model for 2 species (*Geranium sylvaticum* and *Potentilla crantzii*; Table 3A and 3B), and to linear models for the other 3 species *(Linum catharinum*, *Silene acaulis*, and *Veronica fruticans*; Table 3A and 3B). For the rest of the species, excepting *Potentilla thuringica* and *Veronica alpina*, AICc values showed that linear models were better, whereas RRMSE pointed to L&G as the best model (Table 3A and 3B). Again, residual analysis revealed a better fit for the linear models, as most of the L&G models showed non-random linear patterns in the residual plots. The S&F model only performed better for one species (*Potentilla thuringica*; Table 2).

Since the linear models performed generally better than the S&F or L&G models, we shall refer to the results of the linear models in the following sections.

### Trade-off at the fruit/infructescence level and factors related to its intensity

**Fig 1:**
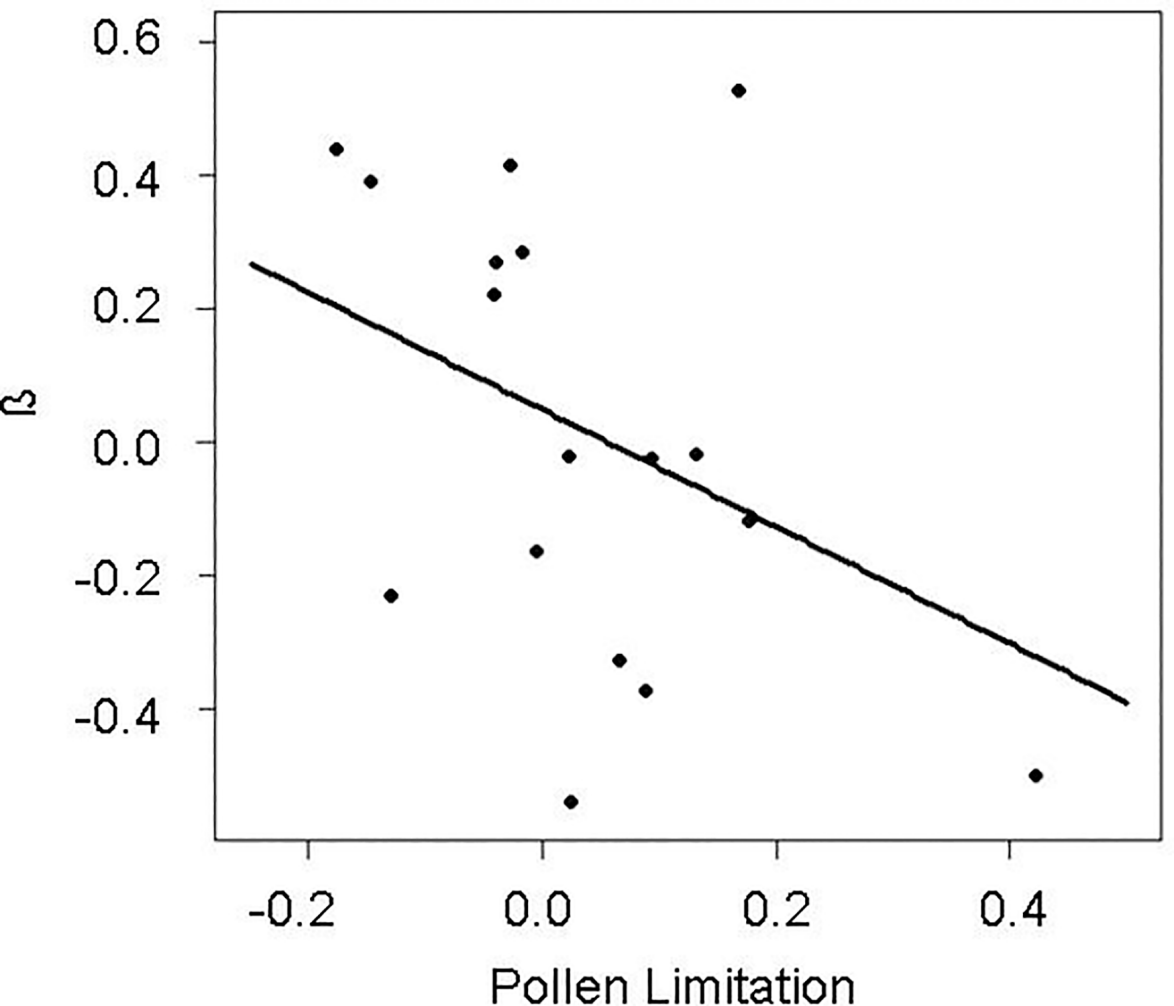
Seed number/size relationship and pollen limitation. Partial residual plot showing the relationship between the standardised slope (β) for the relationship between seed number and size and the pollen limitation index (PL) in the alpine community. PL was calculated as 1- seed set of open pollinated plants / seed set of hand-pollinated plants (see [36] for details), and increases as the value of the index increases. Lines represent the estimates of the best model, and dots represent the partial residuals of the model (sampling units are plant species).

In the lowland community, we found two significant negative relationships between seed number and weight at the fruit level after controlling for multiple testing (Table 4A). At the infructescence level, the only significant relationship found after controlling for multiple testing was positive (Table 4A). In the alpine community, of the five significant slopes at the fruit level, four were negative (Table 4B), while one was positive (Table 4B). At the infructescence level, the only significant relationship was positive. Therefore, overall we found that six of the seven significant relationships between seed number and weight at the fruit level were negative, while the two significant relationships found at the infructescence level were positive. According to this, the best PGLS model showed that the only studied factor affecting the slope of the relationship in the lowland community was fruit organization. The slopes were greater (i.e., more positive) in species with one-seeded fruits aggregated in infructescences compared to single-fruited ones (0.110 ± 0.085 vs. -0.104 ± 0.092, respectively; *F*_1, 20_ = 14.25, *P* = 0.001). This best model showed a λ = -0.66, indicating moderate negative phylogenetic autocorrelation. The best PGLS model for the alpine community showed that as species’ pollen limitation increased, the seed number/size trade-off became more intense, i.e., the slopes were more strongly negative (*F*_1, 16_ = 9.39, *P* = 0.007; Fig 1), while none of the other studied variables appeared in this best model (note that in this community, fruit organization could not be tested as a predictor variable). The low λ value found (λ = -0.16) indicates the large phylogenetic independence of species’ traits from their shared evolutionary history.

**Table 4:** Results of the linear models testing the relationship between seed number and seed weight at the fruit/infructescence level. A) the lowland and B) the alpine communities. F: fruit; I: infructescence; *n*: sample size, *ß*: standardized slope. q* is the level of significance following the Benjamini-Hochberg [41] procedure for controlling the false discovery rate in multiple testing. The null hypothesis (β = 0) is rejected when p> = q* (marked in bold).

Community	Unit	Species	*n*	*ß*	*df*	*F*	*P*	q*
A) Lowland	F	*Fragaria vesca*	39	-0.0382	1;37	0.06	0.816	0.05
		*Galium boreale*	16	-0.1538	1;14	0.39	0.544	0.035
		*Galium mollugo*	78	0.0640	1;76	0.32	0.573	0.040
		*Geranium sylvaticum*	15	0.0907	1;13	0.12	0.730	0.044
		*Geum rivale*	15	0.3422	1;13	1.99	0.182	0.017
		*Lathyrus linifolius*	10	0.2872	1;8	0.90	0.371	0.029
		*Linum catharinum*	71	0.1310	1;69	1.24	0.270	0.025
		*Lotus corniculatus*	16	-0.1989	1;14	0.66	0.430	0.033
		*Polygala vulgaris*	48	-0.0823	1;46	0.33	0.570	0.038
		*Potentilla crantzii*	14	-0.8159	1;12	27.87	**0.000**	0.004
		*Potentilla erecta*	50	-0.3612	1;48	7.50	0.009	0.008
		*Potentilla thuringica*	35	-0.1996	1;33	1.45	0.236	0.018
		*Prunella vulgaris*	24	-0.6432	1;22	16.92	**0.000**	0.004
		*Ranunculus acris*	22	-0.2392	1;20	1.34	0.261	0.023
		*Vicia cracca*	23	0.0562	1;21	0.07	0.790	0.048
	I	*Anthyllis vulneraria*	23	-0.091	1;21	0.19	0.666	0.042
		*Centaurea jacea*	54	0.1845	1;52	1.91	0.173	0.015
		*Centaurea scabiosa*	24	0.5609	1;22	11.02	**0.003**	0.006
		*Knautia arvensis*	13	-0.2375	1;11	0.78	0.397	0.031
		*Leucanthemum vulgare*	76	-0.1031	1;74	0.82	0.369	0.027
		*Pilosella lactucella*	36	0.3207	1;34	4.06	0.052	0.013
		*Pilosella officinarum*	32	0.0624	1;30	0.12	0.731	0.046
		*Pilosella pubescens*	25	0.4308	1;23	5.71	0.025	0.010
		*Trifolium pratense*	15	0.2977	1;13	1.46	0.248	0.021
B) Alpine	F	*Astragalus alpinus*	35	-0.084	1;33	0.25	0.620	0.042
		*Bartsia alpina*	46	-0.4346	1;44	10.74	**0.002**	0.006
		*Campanula rotundifolia*	27	0.4479	1;25	6.65	**0.016**	0.017
		*Cerastium alpinum*	28	-0.4797	1;26	8.38	**0.008**	0.011
		*Dryas octopetala*	39	0.2281	1;37	2.14	0.152	0.025
		*Gentiana nivalis*	40	0	1;38	0.02	0.903	0.044
		*Geranium sylvaticum*	19	0.2780	1;17	1.59	0.224	0.031
		*Parnassia palustre*	73	0.3014	1;71	5.31	0.024	0.019
		*Pinguicula vulgaris*	16	-0.3027	1;14	1.14	0.304	0.036
		*Potentilla crantzii*	55	-0.1887	1;53	2.03	0.160	0.028
		*Ranunculus acris*	42	0.0004	1;40	0.00	0.998	0.05
		*Saxifraga aizodes*	15	0.4523	1;13	3.67	0.077	0.022
		*Silene acaulis*	42	-0.4573	1;40	11.11	**0.002**	0.006
		*Veronica alpina*	52	-0.3461	1;50	7.08	**0.010**	0.014
		*Veronica fruticans*	15	0.2884	1;13	1.33	0.269	0.033
	I	*Erigeron uniflorus*	47	-0.1432	1;45	0.91	0.346	0.039
		*Leontodon autumnalis*	45	0.0072	1;43	0.00	0.962	0.047
		*Saussurea alpina*	57	0.3491	1;55	7.91	**0.007**	0.008

### Trade-off at the plant level

At the plant level, none of the tested relationships between seed number and size were significant either in the lowland (Table 5A) or in the alpine community (Table 5B) after controlling for multiple testing.

**Table 5:** Results of the linear models testing the relationship between seed number and seed weight at the plant level. A) the lowland and B) the alpine communities. *n*: sample size, β: standardized slope. q* is the level of significance following the Benjamini-Hochberg [41] procedure for controlling the false discovery rate in multiple testing. The null hypothesis (β = 0) is rejected when p> = q*.

Community	Species	*n*	*ß*	*df*	*F*	*P*	q*
A) Lowland	*Centaurea jacea*	19	0.0367	1;17	0.03	0.875	0.050
	*Fragaria vesca*	12	-0.2710	1;10	0.95	0.352	0.020
	*Geranium sylvaticum*	11	-0.4268	1;9	2.45	0.152	0.010
	*Geum rivale*	15	-0.0452	1;13	0.03	0.864	0.045
	*Knautia arvensis*	15	-0.1824	1;13	0.52	0.485	0.030
	*Linum catharinum*	15	0.3575	1;13	2.20	0.162	0.015
	*Pilosella pubescens*	12	0.2626	1;10	0.89	0.368	0.025
	*Polygala vulgaris*	14	0.3840	1;12	2.42	0.146	0.005
	*Potentilla thuringica*	15	-0.1723	1;13	0.46	0.510	0.035
	*Ranunculus acris*	14	-0.0905	1;12	0.12	0.740	0.040
B) Alpine	*Astragalus alpinus*	15	-0.2500	1;13	1.00	0.335	0.019
	*Geranium sylvaticum*	15	-0.2487	1;13	0.99	0.338	0.025
	*Potentilla crantzii*	17	-0.5871	1;15	8.94	0.009	0.006
	*Saussurea alpina*	18	-0.0305	1;16	0.02	0.898	0.044
	*Saxifaga aizodes*	15	0.1270	1;13	0.25	0.628	0.038
	*Silene acaulis*	10	-0.0347	1;8	0.01	0.915	0.050
	*Veronica alpina*	13	-0.4559	1;11	3.41	0.092	0.013
	*Veronica fruticans*	15	-0.2369	1;13	0.89	0.362	0.031

### Trade-off at the inter-specific level

**Fig 2:**
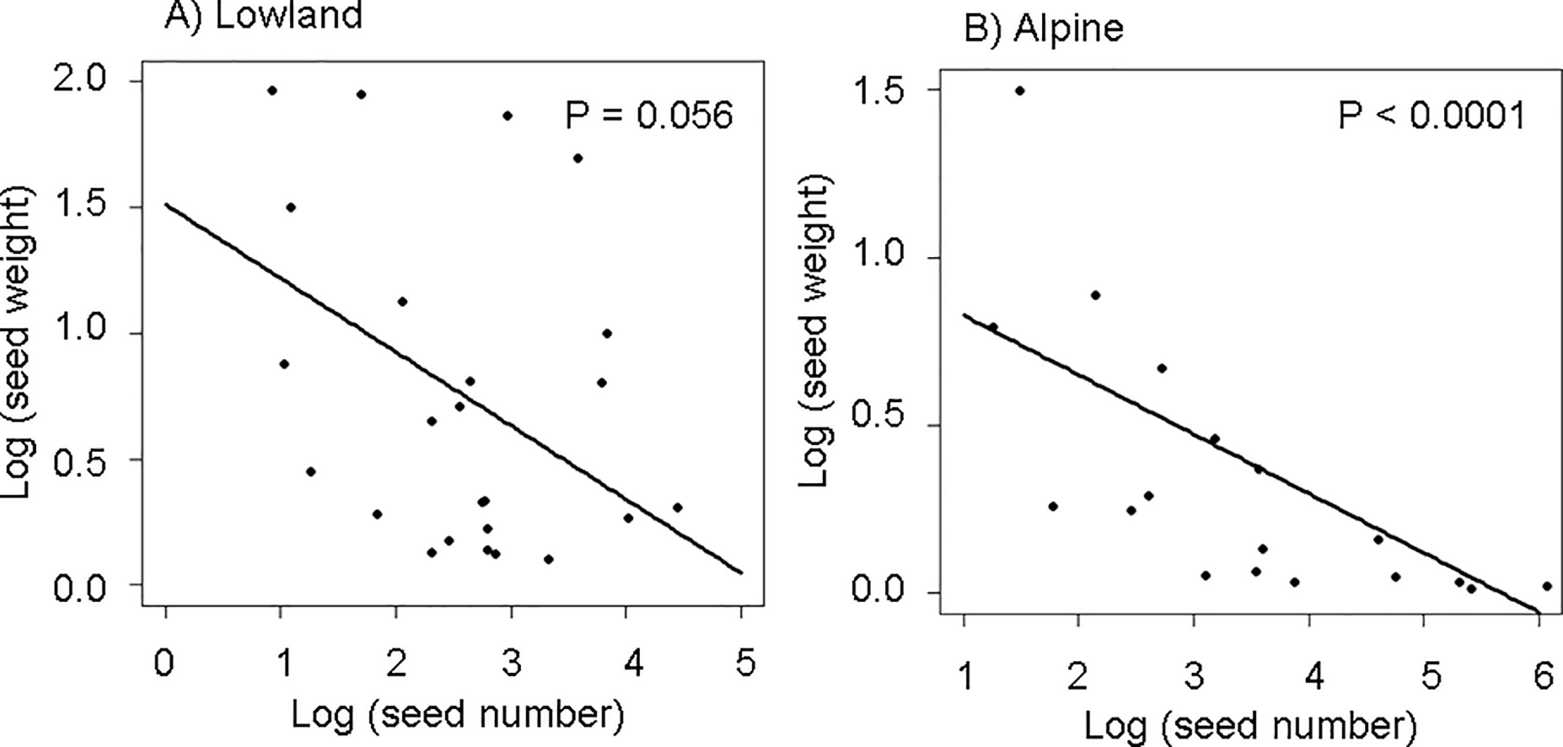
Inter-specific seed number/size trade off. Partial residual plot showing the inter-specific relationship between the log-transformed mean seed number and log-transformed mean seed weight for A) the Lowland community and B) the Alpine community, as estimated by the best model. Dots represent the partial residuals of the model (sampling units are plant species).

In both communities, we found that species with heavier seeds had fewer seeds per fruit/infructescence than those with lighter seeds (Fig 2), although in the lowland community the relationship was only marginally significant (*F*_1, 22_ = 4.06, *P* = 0.056 and *F*_1, 16_ = 19.03, *P* < 0.0001, for Ryghsetra and Finse respectively; Fig 2A and 2B). λ was very close to 1 in both models (0.98 and 1.13 for the lowland and the alpine communities, respectively), suggesting that these traits covary in direct proportion to their shared evolutionary history.

## Discussion

In this study we have shown that, when testing the relationship between seed number and size, the classical linear model performs better for most species and structural levels than the models proposed by Smith and Fretwell [6] and Lescourret and Genard [22]. Although it is usually assumed that a trade-off between seed number and size exists at the intra-specific level, we found a large variability in the relationships at the intra-specific level, with negative, positive, and even an absence of relationships appearing between both variables, depending on the species. However, there were, in general, more negative relationships in single fruits, and more positive relationships in infructescences with one-seeded fruits. According to this, fruit organization (single fruits vs. infrutescences with one-seeded fruits) was the variable with most influence upon the intra-specific seed number/size trade-off in the lowland community. In the alpine community, however, increased pollen limitation decreased the trade-off between seed number and size. In general, we failed to find any evidence for trade-off at the plant level. At the inter-specific level, the species with fewer seeds produced heavier seeds in both communities. However, although this relationship was significant in the alpine community, it was only marginally significant in the lowland community.

### Statistical vs. S&F and L&G models to test the relationship between seed number and size

Intra-specific trade-offs are commonly assumed to drive the relationship between seed number and size, and are generally tested by including the variable seed number in linear models explaining seed mass [3, 8, 16, 18, 19]. We hypothesised that this might not be the best method for testing the trade-off because it is not functionality-based. Instead, we argued that it might be more appropriate to test this trade-off by using the old formulation of Smith and Fretwell [6] or the model proposed by Lescourret and Génard ([22], L&G model). Despite its low prevalence in the literature, the L&G model takes into account the modularity of plants and the hierarchical nature of resource allocation processes, and it has been claimed to fit well with several wild and crop species [22]. The results showed that the commonly used linear models performed better for almost every species and level, with the exception of one species at the fruit level (Table 2), and three at the plant level (Table 3). This may be because, in nature, the relationship between seed number and size is often positive [20, 21], while both the S&F and L&G models force the relationship between seed number and size to be negative [6, 22], with decreasing rates that are linear in the S&F model [6] and that can vary from linear to exponential curves in the L&G model [22]. Both the S&F and L&G models are more stringent than linear models in determining the shape of the relationship between seed number and size, as in both of these models the relationship has to be negative. The S&F model is the most stringent, as the relationship is also forced to be linear. In the case of the L&G model, an additional constraint lies in the fact that model fit is to a great extent affected by the values used to determine the asymptote of the relationship between seed number and size. Those values should correspond to theoretical asymptotic values, which by definition are not observable in nature, and would change depending on environmental factors. Our best estimates, then, will usually be the observed values of nmax and Mmax. These values may not necessarily coincide with the theoretical maximum for the study species at those particular conditions, but they will give reasonable fits if they are close to these theoretical asymptotic values. Unfortunately, many factors could cause the plants not to achieve their theoretical maximum; hence, we have no clue of the suitability of this approximation. This introduces an additional source of uncertainty in the estimation of the parameters for the L&G model. Overall, it could be concluded that, as linear models do not account for all the above-mentioned particularities, they provide a much more flexible framework, which allows coping with the variability in the relationship between seed number and size.

The reason why the lack of flexibility results in bad fits of both the S&F and L&G models is the abundance of positive relationships between seed size and number in our data. Hence, the trade-off proposed by [6] does not seem to be driving the relationship between seed number and size in these communities, or at least not in a general way. We envisage two main reasons why this might be so. First, this trade-off is based on the assumption that the amount of resources available for reproduction in an individual is limited and constant at a given moment [6]. However, plants can vary the proportion of resources allocated to reproduction as a response to environmental conditions [53, 54], and, in fact, the number and size of inflorescences can affect the amount of resources devoted to their development [55]. Photosynthesis of the reproductive structures can also contribute up to 65% of the carbon needed to ripen the fruit ([56], for a review), which means that larger fruits might have proportionally more resources. Second, resources might not be the limiting factor acting on seed production. Seed size and number are also limited by genetics [57] and by environmental factors. For example, under a strong pollen limitation, pollen availability is the limiting factor for seed production. In these cases, the number of seeds produced depends on both the quantity and quality of the pollen received, whereas available resources will be always sufficient to provide all the seeds produced [58, 59, 60].

All in all, both the S&F and L&G models implicitly assume resource availability to be limited and fixed, regardless of unit number, overlooking the influence of additional variables and processes affecting resource availability.

### Trade-off seed number/size at different levels, and variables that explain it at the intra-specific level

At the inter-specific level, seed number and size were negatively related in both communities, although in one community the relationship was only marginally significant. Game theory models analysing the seed number/size trade-off predict that, due to their numerical advantage, smaller-seeded species are able to invade any species mixture, whereas larger seeded species show a competitive superiority over smaller-seeded species during recruitment [32, 33, 61]. Therefore, the inter-specific trade-off is expected to play an important role in both the coexistence of plant species and the diversity and structure of plant communities [28, 29, 32, 33, 34, 35]. Our study adds to the numerous studies indicating a seed number/size trade-off across species [27, 28, 29, 30, 31].

On the contrary, we have shown that the existence of the seed number/size trade-off at the intra-specific level (i.e. at the fruit/infructescence and plant level) also varies greatly among species within the same community, and that all positive, negative or null relationships between seed number and size can be found in some species and at some structural levels. Again, this is in agreement with the results of previous studies at the intra-specific level, which have shown a large variety of responses, from negative [3, 4, 9, 10, 11], to positive relationships [20, 21], as well as the absence of any relationship [15, 16, 17, 18, 19, 20].

Although very little is currently known about the reasons for these variable results at the intra-specific level, some studies have related these variable responses to differences exhibited among individuals in their resource status [62]. Thus, some plants might be resource limited and show seed size-number trade-offs, while other plants with greater access to resources might be able to produce a large number of large seeds [62]. Other studies have shown that the existence of this trade-off within species may be linked to a temporal variation in resource availability and to factors that affect resource allocation within the plant itself. For instance, a trade-off appeared in the masting years in *Buxus balearica* and disappeared in the non-masting years, suggesting that, when resources are scarce, plants fail to appropriately adjust their seed mass and number [23]. A similar pattern has been reported in studies examining seed mass changes over the flowering period [1, 24]. [26] also found that symbiotic plants (*grass-endophyte symbiosis*) showed an overall lower slope in the association between seed number and total reproductive biomass than non-symbiotic plants. We hypothesised that conflicting results for different species might in part be due to the existence of population variables (such as pollen limitation, which will probably vary among studies) and/or species variables that affect the way resources are allocated into seeds (such as fruit organization and the number of seeds/fruit). Particularly, we argued that one-seeded fruit aggregation into infructescences could affect the relationship between seed number and weight because it may affect the way resources are distributed [55, 63, 64]. Similarly, the number of ovules could affect the seed number/size trade-off, as this trait may impose a limit upon the relationship when the number of ovules is very low.

Although we did not find that the number of ovules per flower had any effect on the slope of the relationship between seed number and weight, overall we found different relationships for fruits and infructescences: more negative significant relationships between seed number and size at the fruit level, whereas more positive significant relationships at the infructescence level. These variable results may be related to resources allocation patterns within the plant in different fruit organization structures ([63, 64], and [65], for a review). Thus, our results seem to indicate that within fruits there is stronger competition among seeds for resources than within the clumped infructescences of one-seeded independent fruits. In the infructescences of one-seeded fruits, on the contrary, higher resource availability for the whole infructescence may result in an increase in both seed number and weight. According to this, fruit organization was the most influential variable for the intra-specific slope of the relationship between seed number and size in the lowland community. In the alpine community, however, an increase in species’ pollen limitation increased the trade-off between seed number and weight. This result was contrary to our expectation, which was based on the assumption that resource availability probably would not constrain seed production in pollen-limited species, which might, in turn, have lead to the disappearance of the seed number/size trade-off. However, while pollen limitation could, in theory, affect the seed number/size trade-off (by constraining seed number before seed size is affected by resource scarcity), the relationship between the seed number/size trade-off and pollen limitation could also be modulated by a third variable, producing the observed negative correlation. In particular, the availability of soil nutrients and other resources are known to affect pollen number and quality [66]. Perhaps, in the alpine community, the species facing more intense resource limitation would produce not only lower amounts of pollen, but also pollen of a lower quality. As a consequence, high pollen limitation values could appear to be associated with intense seed number/size trade-offs. Alternatively, this third variable may enter into the allocation trade-off itself, so that seed mass, seed number, and survivorship compete for investment. A recent evolutionary model [67] showed that pollen limitation of ovule fertilization could favour larger individual seeds, as a pollen-limited plant could compensate the fitness loss from low seed set by using its resources to increase individual seed mass, and indirectly juvenile survival [67].

## Conclusions

Despite the appeal of theoretically grounded functional models, we found that simple linear models performed better explaining the relationship between seed number and size, because they provide a more flexible framework that allows coping with the variability of this relationship. Our results support the inter-specific seed number/size trade-off reported in other studies, although the relationship was only marginally significant in one of the communities. However, at the intra-specific level, this trade-off is not a general driver of seed production as it is commonly assumed. The fact that this trade-off does not generally govern the relationship between seed number and size may mean that the quantity of resources available for reproduction is not fixed, and/or that other factors not related to resource availability have a stronger effect on this relationship. Moreover, this study shows that the relationship between seed number and size depends on the level at which it is measured, and also to some extent, on species- and population-dependent variables. To our knowledge, our study is the first to identify some of the causes of this intra-specific variation in the seed number/size relationship, as it is fruit organization (single fruits vs. infructescences) and species’ pollen limitation. Studying the variables that influence the seed number/size relationship and the mechanisms behind their influence is necessary to understand the way plants optimise resources to maximise fitness in different contexts.

## Supporting information

S1 Table Data to test the trade-off at the fruit/infructescence level. Number of seeds and mean seed weight for all the individuals of each study species and site, as well as the plot where they were collected.
(CSV)

S2 Table Data to test the trade-off at the plant level. Number of fruits and weight of the total seed production per fruit for all the individuals of each study species and site.
(CSV)

S3 Table Data to evaluate the factors related to the intensity of the trade-off at the fruit/infructescence level, and to test the trade-off at the inter-specific level. For each study species and site, fruit organization, the number of ovules per flower, pollen limitation indices, the standardized slope for the trade off seed number/size, as well as the mean number of seeds per fruit/infructescence and mean number of weight per seed, are given.
(CSV)
